# Supplementary material for: Fluorescence Labeling of Cellulose Nanocrystals—A Facile and Green Synthesis Route
Source: Polymers (Basel). 2022 Apr 29;14(9):1820. doi: 10.3390/polym14091820 (PMC9099464; doi:10.3390/polym14091820)
Supplement: Supplementary file 1 [file polymers-14-01820-s001.zip › polymers-1679129-supplementary.pdf]

Supplementary Information

Fluorescence labeling of cellulose nanocrystals — a facile and green synthesis route

Lorenzo Donato Campora [1,2], Christoph Metzger [3], Stephan Dähnhardt-Pfeiffer [4], Roland Drexel [5], Florian Meier [5] and Siegfried Fürtauer [2,*]

1 Department of Civil and Industrial Engineering, University of Pisa, 56122 Pisa, Italy; l.campora@studenti.unipi.it
2 Fraunhofer Institute for Process Engineering and Packaging IVV, 85354 Freising, Germany
3 Chair of Process Systems Engineering, TUM School of Life Sciences Weihenstephan, Technical University of Munich, 85354 Freising, Germany; christoph.metzger@tum.de
4 Microscopy Services Dähnhardt GmbH, 24220 Flintbek, Germany; spfeiffer@microscopy-consulting.com
5 Postnova Analytics GmbH, 86899 Landsberg am Lech, Germany; roland.drexel@postnova.com; florian.meier@postnova.com
* Correspondence: siegfried.fuertauer@ivv.fraunhofer.de; Tel.: +49-8161-491-526

Table S1: AF4 fractionation method for the separation of the CNC samples.

**AF4 - Method**	
detector flow rate (mL min^{-1})	0.3
slot flow rate (mL min^{-1})	0.2
injection flow rate (mL min^{-1})	0.2
cross flow rate (initial) (mL min^{-1})	1
delay time (min)	2
injection time (min)	6
transition time (min)	0.5

**Elution**	**duration (min)**	**cross flow rate (mL min^{-1})**	**program**
step 1	2	1	constant
step 2	40	1	power (0.2)
step 3	40	0.1	constant
rinse step	15	0	

Table S2: pH values at different steps of the synthesis of CNC-APTES.

	**Time (min)**	**0**	**10**	**20**	**40**	**45**	**225**
**Sample**	Preparation	pH CNC dispersion (1 wt.%)	pH after HCl addition	pH after APTES addition	pH after hydrolysis of APTES	pH after NaOH addition	pH after conden-sation of APTES
**CNC-APTES-1**	Protocol according to [75]	6.5	4.54	10.42	10.42	10.81	10.66
**CNC-APTES-2**	pH establishment during hydrolysis	7.8	1.58	8.93	3.17	10.07	10.04
**CNC-APTES-3**	Optimized protocol (25 °C) for further FITC-functionalization	7.6	1.48	2.46	2.46	10.19	10.11
**CNC-APTES-4**	Increased condensation temperature (40 °C)	7.48	1.42	2.29	2.29	10.12	10.12

Table S3: Concentrations of FITC in washing water (CNC-APTES-FITC-3).

**Washing step (N)**	**Volume supernatant (ml)**	***c* (FITC) (µmol L^{-1})**	**Ratio *c(N)*/*c(N*+1*)***	**Removed amount per step N; *n* (FITC) (µmol)**	**Removed ratio FITC (%) per step N**	**Removed amount *n* (FITC) (µmol) total**	**Removed ratio FITC (%) total**
0	16.175	289.2	1.6	4.68	29.1	4.68	29.1
1	16.3	178.4	3.3	2.91	18.1	7.59	47.3
2	20.15	54.0	3.4	1.09	6.8	8.67	54.0
3	19.63	16.0	3.2	0.31	2.0	8.99	56.0
4	19.79	5.0	3.1	0.10	0.6	9.09	56.6
5	20.38	1.6	2.8	0.03	0.2	9.12	56.8
6	20.86	0.6		0.01	0.1	9.13	56.9

Table S4: Detailed description of IR modes of CNC, CNC-APTES-3 and CNC-ATPES-FITC-3

IR mode	Reference	Wavenumber ν (cm^{-1})	Visible in… CNC	CNC-APTES-3	CNC-APTES-FITC-3
O-H stretching: O(3)H...O(5) intramolecular	[82,84]	3340	x	x	x
O-H stretching: O(6)H...O(3) intramolecular	[82,84]	3272	x	x	x
C-H (>CH-) stretching	[82,83]	2895	x	x	x
Lactone 5-membered-ring	[86]	1775			x
C=O stretching	[82]	1720			x
H-O-H bending: adsorbed water	[71,83]	1648	x	x	x
C-H (CH2-pyranose ring) scissoring	[82-84]	1429	x	x	x
C-H (>CH-) deformation vibration	[71,82-84]	1370	x	x	x
organic sulfate	[82]	1335	x	x	x
CH2 wagging	[84]	1310	x	x	x
organic sulfate: Asymmetric/symmetric SO2 stretching	[82]	1200	x	x	x
C–O–C asymmetric valence vibration	[82,84]	1162	x	x	x
Ring asymmetric valence vibration	[84]	1105	x	x	x
C–O valence vibration mainly from C3–O3H	[84]	1055	x	x	x
C-O-C pyranose ring vibration	[83]	1030	x	x	x
Si-O-Si	[85]	980		x	x
beta-glycosidic linkage between glucose units in cellulose	[83,84]	895	x	x	x
aromatic C-H out-of-plane bending	[82]	875			x
Si-C	[75]	845		x	x
O-H out-of-plane bending	[82-84]	665	x	x	x

Blue: characteristic bands for modified CNC (CNC-APTES-3 and CNC-APTES-FITC-3)

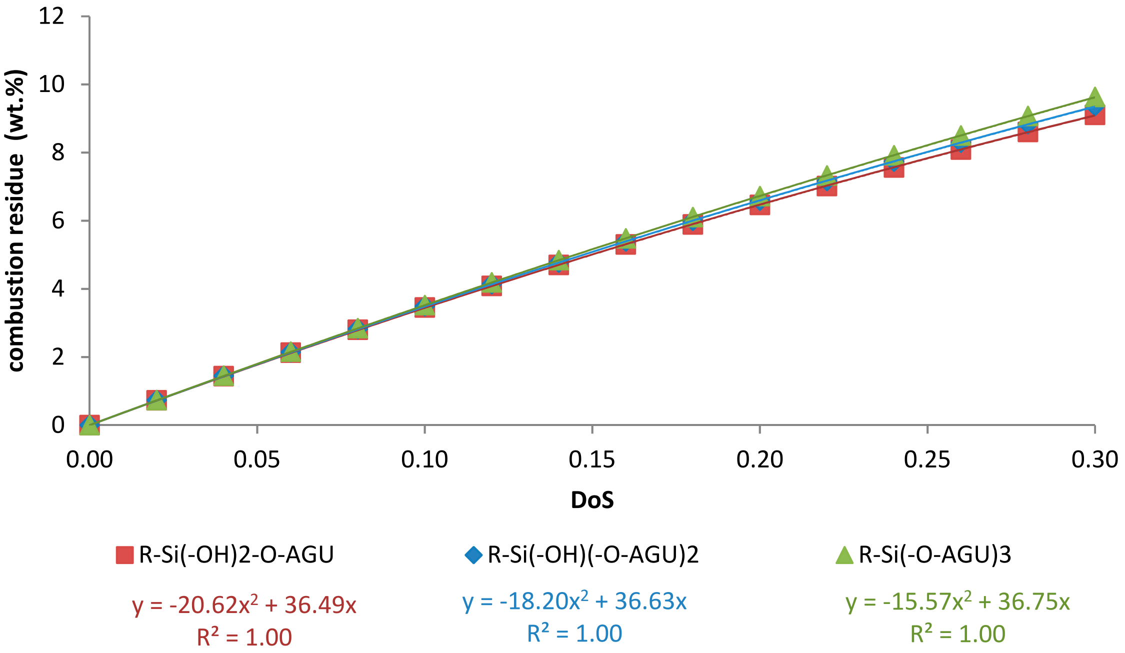

Figure S1: Relation between combustion residue (wt.%) of CNC-APTES and DoS.

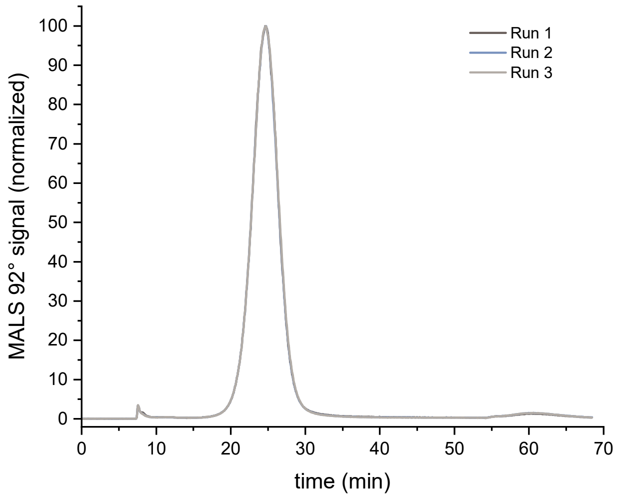

Figure S2: Fractograms of triplicate separation of polystyrene beads (size standards with D = 62 nm ± 4 nm, NanoSphere™ 3060A Thermo Scientific™). Retention time of the peak maximum was used to determine the effective channel height = 331.41 µm ± 0.13 µm.

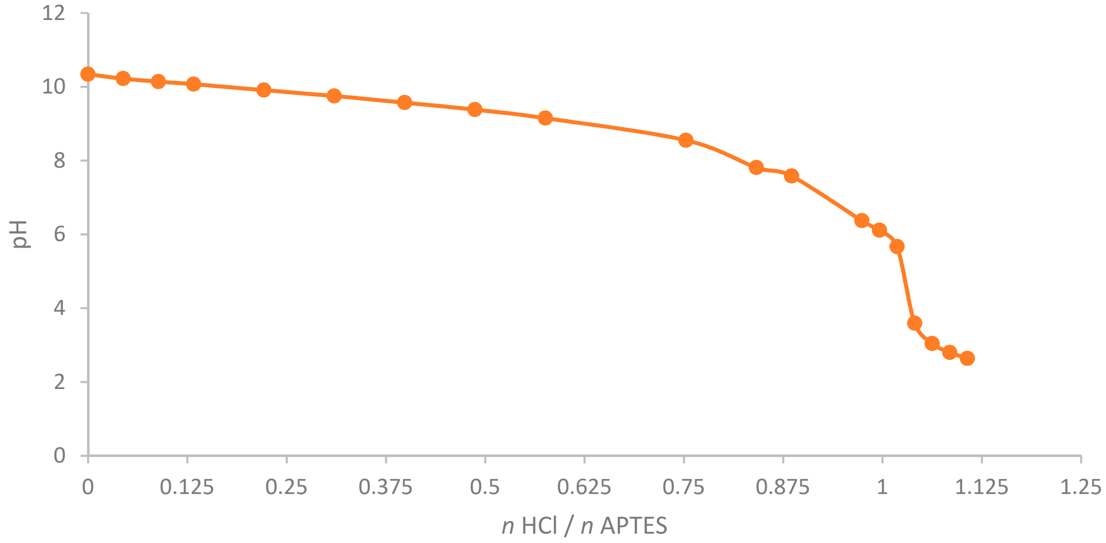

Figure S3 (a) Titration of APTES with HCl 0.5 mol L^{-1}.

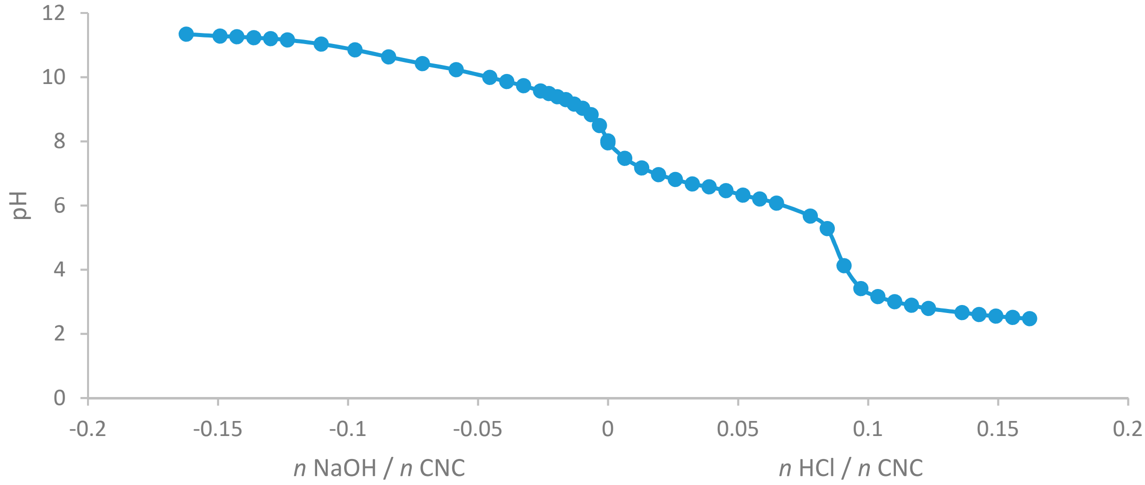

Figure S3 (b) Titration of CNCs with NaOH 0.1 mol L^{-1} (negative x axis) and HCl 0.1 mol L^{-1} (positive x axis).

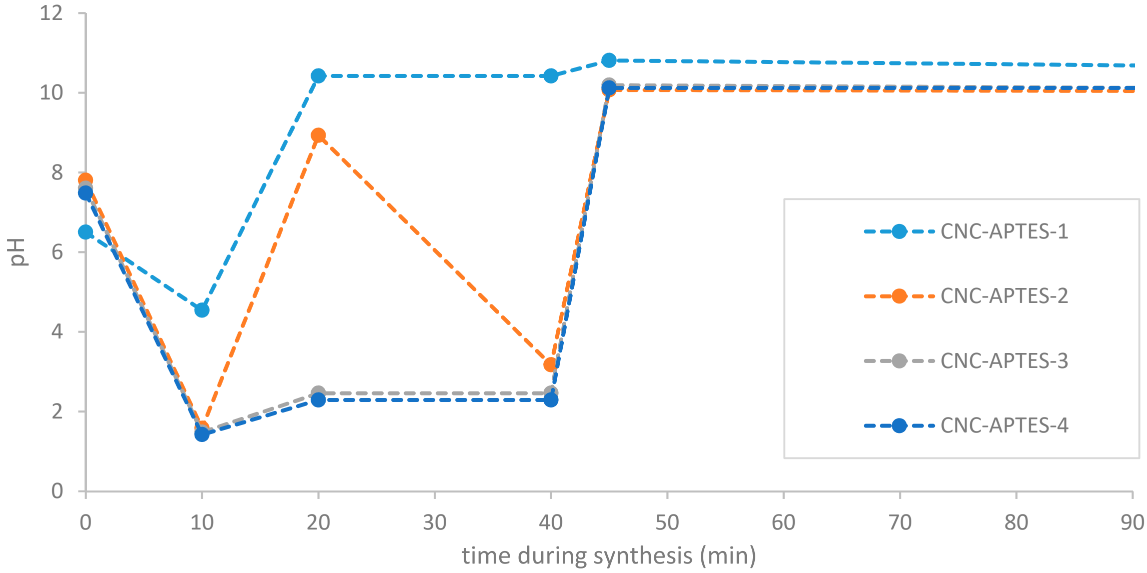

Figure S4: pH values at different steps of the synthesis of CNC-APTES.

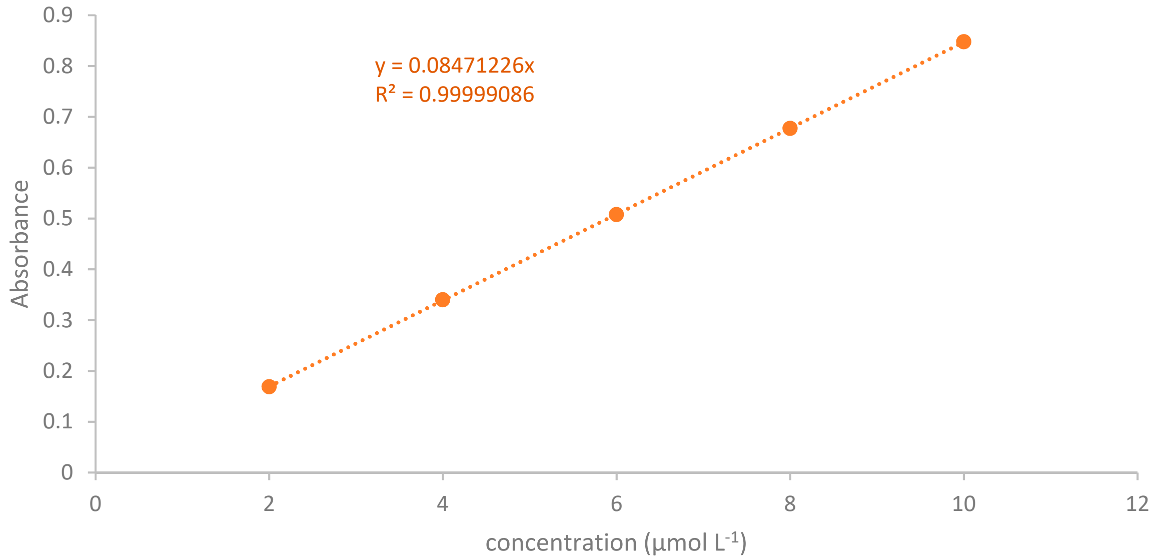

Figure S5: Calibration of FITC in buffer for photometry (at 495 nm).

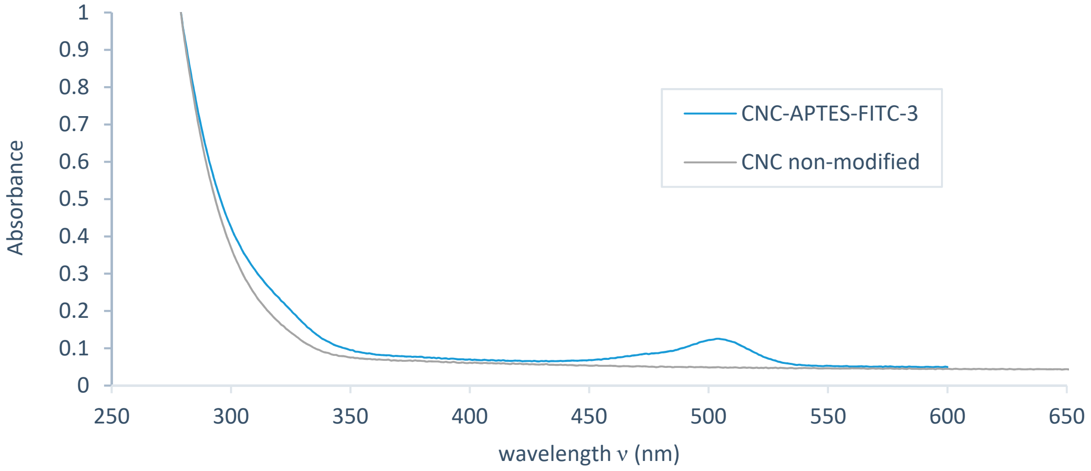

Figure S6: Spectrum of CNC non-modified and CNC-APTES-FITC-3.
